# Identification of differentially expressed genes in uveal melanoma using suppressive subtractive hybridization

**Published:** 2011-05-13

**Authors:** Solange Landreville, Caroline B. Lupien, Francois Vigneault, Manon Gaudreault, Mélissa Mathieu, Alain P. Rousseau, Sylvain L. Guérin, Christian Salesse

**Affiliations:** LOEX/CUO recherche, Centre hospitalier affilié universitaire de Québec, Hôpital du Saint-Sacrement, and Département d’ophtalmologie, Faculté de médecine, Université Laval, Québec, Québec, Canada

## Abstract

**Purpose:**

Uveal melanoma (UM) is the most common primary cancer of the eye, resulting not only in vision loss, but also in metastatic death. This study attempts to identify changes in the patterns of gene expression that lead to malignant transformation and proliferation of normal uveal melanocytes (UVM) using the Suppressive Subtractive Hybridization (SSH) technique.

**Methods:**

The SSH technique was used to isolate genes that are differentially expressed in the TP31 cell line derived from a primary UM compared to UVM. The expression level of selected genes was further validated by microarray, semi-quantitative RT–PCR and western blot analyses.

**Results:**

Analysis of the subtracted libraries revealed that 37 and 36 genes were, respectively, up- and downregulated in TP31 cells compared to UVM. Differential expression of the majority of these genes was confirmed by comparing UM cells with UVM by microarray. The expression pattern of selected genes was analyzed by semi-quantitative RT–PCR and western blot, and was found to be consistent with the SSH findings.

**Conclusions:**

We demonstrated that the SSH technique is efficient to detect differentially expressed genes in UM. The genes identified in this study represent valuable candidates for further functional analysis in UM and should be informative in studying the biology of this tumor.

## Introduction

Uveal melanoma (UM) is a malignant tumor that arises from neural crest-derived melanocytes of the uveal tract of the eye [1]. It is the most prevalent primary cancer of the eye with an annual frequency of 4–7 cases per million of population in North America [1]. This ocular tumor not only has the capacity to destroy vision but can also metastasize and ultimately cause death in up to half of patients diagnosed with this type of cancer. Despite chemotherapy, the metastatic disease is fatal, usually within a few months of diagnosis [1].

Carcinogenesis occurs as an accumulation of molecular events involving disruption of cell cycle and apoptotic control, as well as increased aneuploidy leading ultimately to malignant transformation and dissemination of tumor cells. In the past decade, many details of the pathogenesis of UM have emerged, e.g., the gene-expression signatures with prognostic significance, as well as guanine nucleotide-binding proteins alpha-q and alpha 11 (*GNAQ/GNA11*) and BRCA1 associated protein-1 (*BAP1*) mutations [2-5]. Despite these discoveries, a better insight into tumor progression of UM primary tumors remains of utmost importance to identify new therapeutic targets. One way to better understand the malignant transformation of cells is to determine which genes are differentially expressed between primary tumors and normal melanocytes.

The suppressive subtractive hybridization (SSH) technique, a combination of subtraction and kinetic enrichment coupled to subsequent amplification, increases the representation of rare mRNAs, which enables to compare two different populations of mRNAs and to obtain clones of genes that are differentially expressed in the population of interest [6,7]. This technique has proven to be useful in identifying tissue-specific and less abundant transcripts as it can achieve over a 1,000 fold enrichment for differentially expressed cDNA populations [6,8]. Here, we describe the identification of 73 genes differentially expressed in primary UM using the SSH technique. Some of these genes are likely involved in malignant transformation and should be informative in studying the biology of UM.

## Methods

This study followed the principles of the Declaration of Helsinki and was approved by our institutional human experimentation committee. Written informed consent was obtained from the enucleated subjects.

### Tissue collection and cell culture

The TP31 cell line is derived from a mixed epitheloid-spindle primary UM tumor of a 62-year-old patient that died of liver metastases (Table 1) [9]. This cell line does not have mutations in *GNAQ* or *GNA11* and was cultured in DMEM/F12 medium (Gibco BRL, Burlington, ON) supplemented with 10% FBS (Gemini; NorthBio, Toronto, ON) under 5% CO_2_. Normal uveal melanocytes (UVM) were grown from human donor eyes provided by the Banque d’Yeux Nationale (CHUL, Québec, QC), according to the procedure described by Hu et al. [10]. Samples of UM primary tumors were collected at the time of enucleation (Table 1) and were either immediately stored at −80 °C in Tri-Reagent for RNA extraction (Sigma-Aldrich, Oakville, ON) or grown in tissue culture for less than 9 passages [9]. Tumors were classified according to a modification of the Callender’s classification [11].

**Table 1:** Clinicopathological characteristics and survival data of uveal melanoma cases.

**#**	**Age, Sex**	**Size**	**Last status**	Follow-up* (months)	**Pathology**
TP31	62, M	Large	Dead of metastasis	42	Mixed
1	69, M	Large	Alive without metastasis	104	Mixed
2	33, M	Large	Alive without metastasis	104	Spindle
3	45, M	Medium	Alive without metastasis	81	Spindle
4	34, F	Medium	Alive without metastasis	103	Spindle
5	69, M	Small	Alive without metastasis	57	Spindle
6	46, M	Large	Dead of metastasis	18	Epithelioid

*Follow-up: period from enucleation until patient death or last visit.

### Suppressive subtractive hybridization (SSH)

Total RNA derived from both the TP31 cell line and UVM (pool of 18 donors) was extracted with the RNeasy kit (Qiagen, Mississauga, ON) and mRNA was isolated with the Oligotex mRNA kit (Qiagen) according to the manufacturer’s instructions. cDNA was synthesized using the SMART PCR cDNA Synthesis kit (Clontech Laboratories, Mountain View, CA). Two SSH libraries were then performed between the TP31 cell line and UVM (a forward subtraction named “TP31 cell line subtracted library” and a reverse one named “UVM subtracted library”) using the PCR-Select cDNA Subtraction kit (Clontech Laboratories) according to the procedure described by Diatchenko et al. [7]. In the forward subtraction, the TP31 cell line was used as the tester and UVM as the driver (TP31 cell line subtracted library, which corresponds to upregulated genes in the TP31 cell line) whereas the UVM were used as the tester and the TP31 cell line as the driver in the reverse subtraction (UVM subtracted library, which corresponds to downregulated genes in the TP31 cell line). To evaluate the efficiency of the cDNA subtraction, the expression level of actin and endothelin receptor type B (*EDNRB*; Table 2) was monitored by RT–PCR in subtracted cDNA and unsubtracted cDNA. Aliquots of subtracted and unsubtracted cDNAs were removed from each reaction after 18, 23, 28, and 33 cycles and compared by agarose gel electrophoresis.

**Table 2:** Sequence of forward and reverse primers used for PCR amplification.

**Gene**	**Forward primer (5′-3′)**	**Reverse primer (5′-3′)**	**Expected PCR product size (bp)**
*Actin*	TGTCCACCTTCCAGCAGATGT	CACTCCCAGGGAGACCAAAA	609
*ANLN*	CCAAGTCCTGTGTCCTCA	TGTCCCTCACAACTTTTAGCA	643
*ANP32E*	CGCGCTAGTGTGTGGACAAG	CGGCGCTTCATTATCCTCCT	700
*CKAP5*	CAGTGAGTGGTTGAAACTGCC	CTCCAGGGCCTCTTTTCTC	895
*CTSK*	ACCCCGGTTCTTCTGCACAT	GCCGAGGTACCCCTGTCTCA	306
*DTL*	CTTGGCGTCCTGAGAAATGG	TGGAAATCCACAGAAGGAGCA	614
*EDNRB*	CCAACATGTGGCCCAGCCTA	TGAGGTGGGGTTGGAGGAAA	231
*MTAP*	AGTAGCATGGCTGCCCAGGA	CCCTCCACCCTTTATTGTTGC	310
*PPP3CA*	AGGCAATTGATCCCAAGTTGT	GGGGTAGAGAATTTTCAAGGC	409
*TSPYL5*	AGATGCAAGGGAAAGGAAGCA	CTCGGACCCCATGTGTCCAT	323
*TYRP1*	ACCGCTGTGGCTCATCATCA	TCCCCGTTGCAAAATTCCAG	603

### Cloning, differential screening, sequencing and analysis of the subtracted cDNAs

The PCR products of the SSH libraries were purified (NucleoSpin Extract kit; Clontech Laboratories) and then inserted into the T/A cloning vector pGEM-T Easy (Promega, Madison, WI). Individual transformants carrying subtracted cDNA fragments were isolated from white colonies and used for differential screening (PCR-Select Differential Screening kit; Clontech Laboratories) to eliminate false positives, according to the manufacturer’s instructions. PCR fragments of the positive clones were isolated with QIAquick PCR Purification kit (Qiagen) and then sequenced using Nested PCR Primers 1 and 2R (Clontech Laboratories) with an automated DNA sequencer (ABI Prism model 3900; Applied Biosystems, Foster City, CA). DNA sequencing of positive clones was performed by the Plateforme de séquençage et de génotypage des génomes at Université Laval (Québec, QC). The inserted sequences were examined for similarities to human genes with the NCBI BLAST program. Poly (A)^+^, vector sequences, and sequences with many ambiguities were manually removed from the sequence data. A sequence was considered significant to a database entry when an aligned region was more than 95% identical over the entire cDNA length.

### Microarray gene expression profiling

Gene expression profiling was performed as previously reported [12] using HumanHT-12 v3 Expression BeadChip arrays (48,804 probes; Illumina, San Diego, CA). Data were analyzed using the ArrayStar v3.0 software (DNASTAR, Madison, WI).

### Semi-quantitative RT–PCR

Total RNA derived from the TP31 cell line, as well as from UVM was extracted with the RNeasy kit (Qiagen) as described above. Total RNA derived from uncultured UM primary tumors was isolated using Tri-Reagent (Sigma-Aldrich, Oakville, ON) according to the manufacturer’s instructions. Reverse transcription was performed using random hexamer primers following manufacturer’s protocol for synthesis of first strand cDNA (MBI Fermentas, Burlington, ON). Primers (Table 2) were designed using the GenBank database (NCBI, Bethesda, MD). Semi-quantitative RT–PCR was performed as described previously [13] using the QuantumRNA 18S Internal standards protocol (Ambion, Austin, TX) according to the manufacturer’s instructions.

### SDS–PAGE and western blot

Protein extraction and SDS–PAGE/western blot were performed as described previously [14]. Proteins were separated on 10% polyacrylamide gels, transferred onto a nitrocellulose membrane (Biorad, Hercules, CA) and incubated with antibodies directed against β-actin (loading control; mouse, 0.025 µg/ml; Cedarlane, Hornby, ON), anillin (ANLN; rabbit, 1.0 µg/ml; kindly provided by Dr. Peter A. Hall, Queens University, Belfast, Ireland), protein phosphatase 3 catalytic subunit alpha isoform (PPP3CA; rabbit, 1.0 µg/ml; Chemicon, Temecula, CA) and tyrosinase-related protein 1 (TYRP1; rabbit, 0.08 µg/ml; Santa Cruz Biotechnology, Santa Cruz, CA). The blots were visualized using the Fluor-S Max System (Biorad, Hercules, CA) after incubation with peroxidase-conjugated secondary antibodies.

## Results

### Evaluation of subtraction efficiency

**Figure 1:**
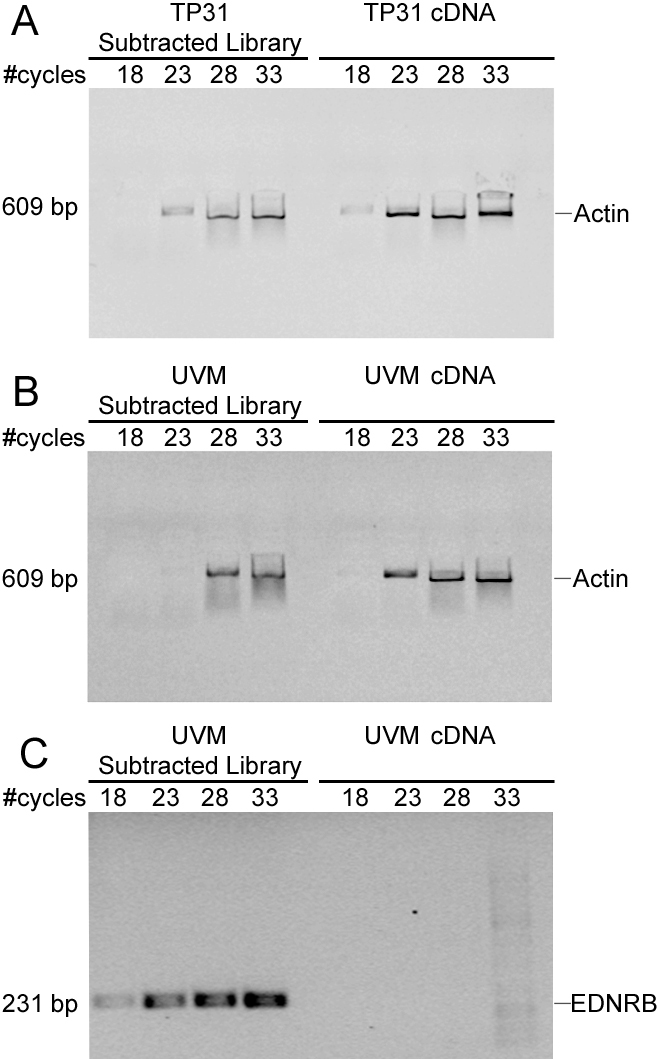
Evaluation of the subtraction efficiency by PCR using the housekeeping gene actin and the melanocyte marker *EDNRB*. **A**: Reduction of actin expression in the TP31 subtracted library compared to unsubtracted TP31 cDNA. **B**: Reduction of actin expression in the UVM subtracted library compared to unsubtracted UVM cDNA. **C**: Enrichment of *EDNRB* expression in the UVM subtracted library compared to unsubtracted UVM cDNA. Samples were taken after 18, 23, 28, and 33 PCR cycles.

Successful SSH should decrease housekeeping gene transcripts abundance and enrich tissue-specific gene transcripts [6]. A reduction of actin mRNA expression can be observed in the TP31 subtracted library compared to the unsubtracted TP31 cDNA. Indeed, the actin amplicon can be observed after 23 cycles of amplification in the subtracted library compared to 18 cycles for the unsubtracted population (Figure 1A). A similar reduction of actin mRNA expression was observed in the UVM subtracted library (Figure 1B). As a positive control for the enrichment of differentially expressed genes, *EDNRB,* a melanocytic lineage marker was amplified. *EDNRB* amplicon can be observed after 18 cycles in the UVM subtracted library compared to 33 cycles for the unsubtracted population (Figure 1C). These data demonstrate successful subtractions, with significant reduction of actin abundance and significant enrichment of *EDNRB*, 5 cycles corresponding roughly to a 20-fold cDNA concentration difference.

### Comparison of mRNA profiles between uveal melanoma and normal uveal melanocytes using SSH

The analysis of the TP31 cell line subtracted library (UM upregulated genes) showed that 58 positive clones corresponded to 37 genes when taking the redundancy into account (Table 3). 57% of these genes were previously associated with cancer (highlighted in bold in Table 3). The most highly represented genes in this subtracted library were anillin (*ANLN*) and the cytoskeleton associated protein 5 (*CKAP5*). Analysis of the UVM subtracted library (UM downregulated genes) showed that 54 positive clones corresponded to 36 genes when taking the redundancy into account (Table 4). Among these genes, 55% were previously found to be downregulated in cancer (highlighted in bold in Table 4). The most highly represented genes in the UVM subtracted library were actin gamma 1 (*ACTG1*), alpha-2-macroglobuline (*A2M*), cathepsin K (*CTSK*), and proteolipid protein 1 (*PLP1*). Comparison with microarray data of the previously published transcriptome of UVM [12] confirmed an upregulation of 70% of the genes identified in the TP31 cell line subtracted library and a downregulation of 81% of the genes identified in the UVM subtracted library (Table 3 and Table 4, last column). The most represented biologic processes were associated to cell division and metabolism for the upregulated genes, while they were linked to differentiation and G-protein signaling for the downregulated genes.

**Table 3:** Upregulated genes from the TP31 cell line subtracted library.

**Gene**	**Chromosomal location**	**GenBank accession #**	**Biological process**	**Redundancy**	Microarray fold-change UM/UVM*
**Acidic (leucine-rich) nuclear phosphoprotein 32 member E (ANP32E)**	**1q21.2**	**NM_030920**	**Apoptosis**	**1**	**1.59**
Alkylglycerone phosphate synthase (AGPS)	2q31.2	NM_003659	Lipid metabolism	2	2.85
**Anillin actin binding protein (ANLN)**	**7p14**	**NM_018685**	**Cell division**	**4**	**2.69**
ATP synthase H^+^ transporting mitochondrial F0 complex subunit G (ATP5L)	11q23.3	NM_006476	ATP synthesis	1	11.20
**Calumenin (CALU)**	**7q32.1**	**NM_001219**	**Calcium ion binding**	**2**	**1.43**
Chromosome 6 open reading frame 211 (C6orf211)	6q25.1	NM_024573	Unknown	2	1.72
Chromosome 6 open reading frame 62 (C6orf62)	6p22.3	NM_030939	Unknown	1	0.43
Chromosome 7 open reading frame 64 (C7orf64)	7q21.2	NM_032120	Unknown	2	1.53
**Cytoskeleton associated protein 5 (CKAP5)**	**11p11.2**	**NM_014756**	**Cell division**	**5**	**4.13**
**Denticleless homolog (DTL)**	**1q32**	**NM_016448**	**Protein ubiquitination**	**1**	**3.79**
Family with sequence similarity 126 member B (FAM126B)	2q33.1	NM_173822	Unknown	1	1.28
Family with sequence similarity 35 member A (FAM35A)	10q23.2	NM_019054	Unknown	1	1.02
Family with sequence similarity 63 member B (FAM63B)	15q21.3	NM_001040450	Unknown	1	0.92
**Glutamate-ammonia ligase (GLUL)**	**1q31**	**NM_002065**	**Cell proliferation**	**1**	**1.65**
HEAT repeat containing 5A (HEATR5A)	14q12	NM_015473	Unknown	2	1.63
**Histone deacetylase 3 (HDAC3)**	**5q31**	**NM_003883**	**Chromatin modification**	**1**	**3.03**
**Human T-cell leukemia virus type 1 binding protein 1 (TAX1BP1)**	**7p15**	**NM_006024**	**Apoptosis**	**1**	**2.03**
**Integrin alpha 6 subunit (ITGA6)**	**2q31.1**	**NM_000210**	**Cell adhesion**	**2**	**1.68**
**Interleukin 1 receptor antagonist (IL1RN)**	**2q14.2**	**NM_173842**	**Inflammatory response**	**5**	**1.71**
KIAA2018	3q13.2	NM_001009899	Unknown	2	1.62
Melanocortin 3 receptor (MC3R)	20q13.2	NM_019888	G-protein signaling	1	1.81
Microphthalmia-associated transcription factor (MITF)	3p14.2	NM_198159	Melanocyte differentiation	2	0.48
**Multiple coagulation factor deficiency protein 2 (MCFD2)**	**2p21**	**NM_139279**	**Vesicule-mediated transport**	**1**	**1.37**
**MutL homolog 3 (MLH3)**	**14q24.3**	**NM_014381**	**DNA repair**	**2**	**1.43**
Oligosaccharyltransferase complex subunit (OSTC)	4q25	NM_021227	Protein glycosylation	1	4.31
**Peroxisome proliferator-activated receptor gamma coactivator 1 (PPARGC1)**	**4p15.1**	**NM_013261**	**Cellular respiration**	**1**	**0.51**
**Pleckstrin homology domain containing family A member 8 (PLEKHA8)**	**7p21-p11.2**	**NM_032639**	**Protein transport**	**1**	**1.38**
Potassium voltage-gated channel, Shab-related subfamily member 1 (KCNB1)	20q13.2	NM_004975	Potassium ion transport	1	1.84
**Proteasome 26S subunit non-ATPase 2 (PSMD2)**	**3q27.1**	**NM_002808**	**Protein ubiquitination**	**1**	**1.40**
**Proteasome maturation protein (POMP)**	**13q12.3**	**NM_015932**	**Proteasome assembly**	**1**	**3.16**
**Protein phosphatase 3 catalytic subunit alpha isozyme (PPP3CA)**	**4q24**	**NM_000944**	**Protein phosphorylation**	**1**	**1.51**
Required for meiotic nuclear division 5 homolog A (RMND5A)	2p11.2	NM_022780	Cell division	1	1.77
**Topoisomerase (DNA) II alpha (TOP2A)**	**17q21-q22**	**NM_001067**	**DNA replication**	**1**	**19.52**
**Translocase of inner mitochondrial membrane 17 homolog A (TIMM17A)**	**1q32.1**	**NM_006335**	**Mitochondrial protein transport**	**1**	**1.63**
**Tyrosine 3-monooxygenase/tryptophan 5-monooxygenase activation protein beta polypeptide (YWHAB)**	**20q13.1**	**NM_003404**	**Ras signal transduction**	**1**	**4.00**
**Vaccinia related kinase 1 (VRK1)**	**14q32**	**NM_003384**	**Protein phosphorylation**	**2**	**6.53**
Zinc finger, HIT-type containing 6 (ZNHIT6)	1p22.3	NM_017953	Ribosome biogenesis	1	3.89

*Considerated validated if the UM/UVM fold-change was >1.5. Genes highlighted in bold were previously shown to be upregulated in cancer.

**Table 4:** Downregulated genes from the UVM subtracted library.

**Gene**	**Chromosomal location**	**GenBank accession#**	**Biological process**	**Redundancy**	Microarray fold-change UM/UVM*
**Actin gamma 1 (ACTG1)**	**17q25.3**	**NM_001614**	**Motility**	**3**	**−86.7**
**A kinase (PRKA) anchor protein 12 (AKAP12)**	**6q25**	**NM_005100**	**G-protein signaling**	**1**	**−1.91**
**Alpha-2-macroglobuline (A2M)**	**12p13.31**	**NM_000014**	**Cytokine transport**	**3**	**−44.4**
Cathepsin K (CTSK)	1q21	NM_000396	Proteolysis	3	−106.5
Chromosome 1 open reading frame 124 (C1orf124)	1q42	NM_032018	DNA repair	2	−0.62
Chromosome 18 open reading frame 32 (C18orf32)	18q21.1	NM_001035005	NF-kappaB cascade regulation	1	−2.69
Dynein cytoplasmic 1 light intermediate chain 2 (DYNC1LI2)	16q22.1	NM_006141	Endosome transport	1	−2.61
**E74-like factor 1 (ets domain transcription factor) (ELF1)**	**13q13**	**NM_172373**	**Transcription**	**1**	**−1.83**
**Endothelin receptor type B (EDNRB)**	**13q22**	**NM_000115**	**Melanocyte differentiation**	**1**	**−38.9**
**Epithelial membrane protein 1 (EMP1)**	**12p12.3**	**NM_001423**	**Epithelial cell differentiation**	**1**	**−148.8**
Formin binding protein 4 (FNBP4)	11p11.2	NM_015308	Unknown	1	−7.82
**Glycoprotein (transmembrane) nmb (GPNMB)**	**7p15**	**NM_002510**	**Melanocyte differentiation**	**1**	**−84.5**
**Guanine nucleotide binding protein alpha inhibiting polypeptide 3 (GNAI3)**	**1p13**	**NM_006496**	**G-protein signaling**	**2**	**−0.82**
**Guanine nucleotide binding protein (G protein) gamma 11 (GNG11)**	**7q21**	**NM_004126**	**G-protein signaling**	**1**	**−6.56**
Heterochromatin protein 1 binding protein 3 (HP1BP3)	1p36.12	NM_016287	Nucleosome assembly	1	−2.37
Importin 7 (IPO7)	11p15.4	NM_006391	Protein transport	2	−0.64
Junction mediating and regulatory protein p53 cofactor (JMY)	5q14.1	NM_152405	Apoptosis	1	−1.68
Leucine rich repeat containing 39 (LRRC39)	1p21.2	NM_144620	Unknown	1	−3.90
**Lysophosphatidic acid receptor 6 (LPAR6)**	**13q14**	**NM_005767**	**G-protein signaling**	**2**	**−20.7**
**Methylthioadenosine phosphorylase (MTAP)**	**9p21**	**NM_002451**	**Polyamine metabolism**	**1**	**−2.55**
**Nerve growth factor receptor associated protein 1 (NGFRAP1)**	**Xq22.2**	**NM_206915**	**Apoptosis**	**1**	**−2.45**
Potassium channel tetramerisation domain containing 18 (KCTD18)	2q33.1	NM_152387	Potassium ion transport	1	−1.33
Potassium inwardly-rectifying channel subfamily J member 13 (KCNJ13)	2q37	NM_002242	Potassium ion transport	2	−21.9
**Proteolipid protein 1 (PLP1)**	**Xq22**	**NM_000533**	**Glial cell differentiation**	**4**	**−5.78**
**Rho-related BTB domain-containing protein 3 (RHOBTB3)**	**5q15**	**NM_014899**	**Retrograde transport**	**1**	**−2.69**
Ribosomal protein S6 kinase polypeptide 1 (RPS6KC1)	1q41	NM_012424	Protein phosphorylation	1	−1.64
SLIT and NTRK-like family member 2 (SLITRK2)	Xq27.3	NM_032539	Axonogenesis	2	−2.51
**SRY (sex determining region Y)-box 4 (SOX4)**	**6p22.3**	**NM_003107**	**Transcription**	**1**	**−1.26**
Subunit of the oligosaccharyltransferase complex homolog B (STT3B)	3p23	NM_178862	Protein glycosylation	2	−1.50
**Transcription elongation factor A (SII)-like 7 (TCEAL7)**	**Xq22.1**	**NM_152278**	**Transcription**	**1**	**−2.82**
Transmembrane emp24 protein transport domain containing 7 (TMED7)	5q22.3	NM_181836	ER transport	2	−1.15
**Testis-specific protein-like 1, y-encoded (TSPYL5)**	**8q22.1**	**NM_033512**	**Nucleosome assembly**	**1**	**−4.02**
**Tyrosinase-related protein 1 (TYRP1)**	**9p23**	**NM_000550**	**Melanocyte differentiation**	**1**	**−397**
**Ubiquitin-conjugating enzyme E2H (UBE2H)**	**7q32**	**NM_003344**	**Protein ubiquitination**	**1**	**−2.32**
WW domain binding protein 5 (WBP5)	Xq22.2	NM_016303	Unknown	1	−2.54
**Zinc finger AN1-type domain 5 (ZFAND5)**	**9q21**	**NM_006007**	**Development**	**2**	**0.61**

*Considerated validated if the UM/UVM fold-change was <-1.5. Genes highlighted in bold were previously shown to be downregulated in cancer.

### Validation of selected up- and downregulated genes by semi-quantitative RT–PCR and western blot

**Figure 2:**
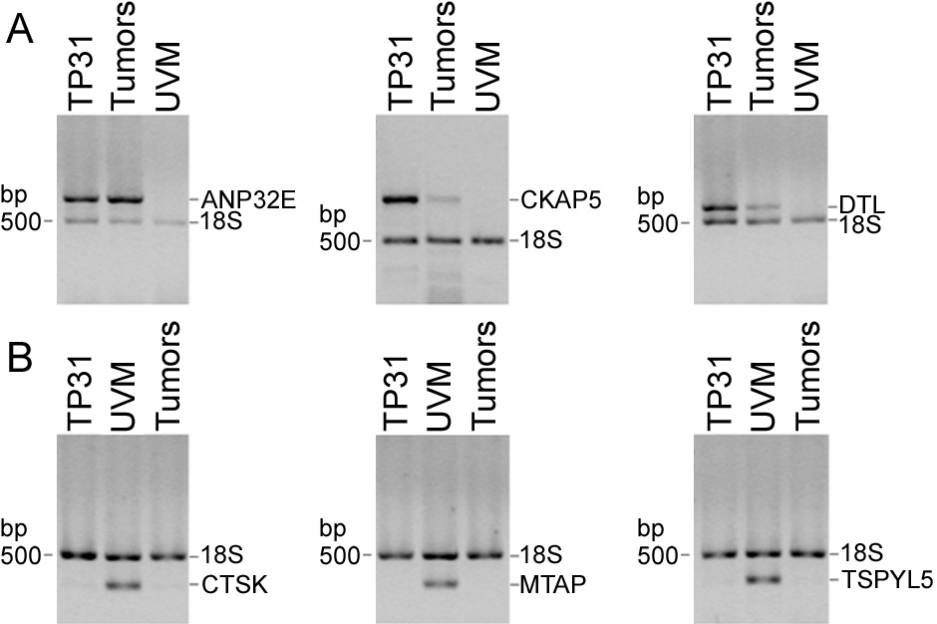
Validation of upregulated and downregulated genes identified in the subtracted libraries. The mRNA expression level of selected genes was measured by semi-quantitative RT–PCR in the TP31 cell line, a pool of RNA from uncultured UM primary tumors (Tumors) and UVM. **A**: Upregulated genes identified in the TP31 subtracted library (*ANP32E*, *CKAP5*, *DTL*). **B**: Downregulated genes identified in the UVM subtracted library (*CTSK*, *MTAP*, *TSPYL5*). The 18S rRNA was used as an internal control of amplification (489 bp). Data are representative of three independent experiments.

**Figure 3:**
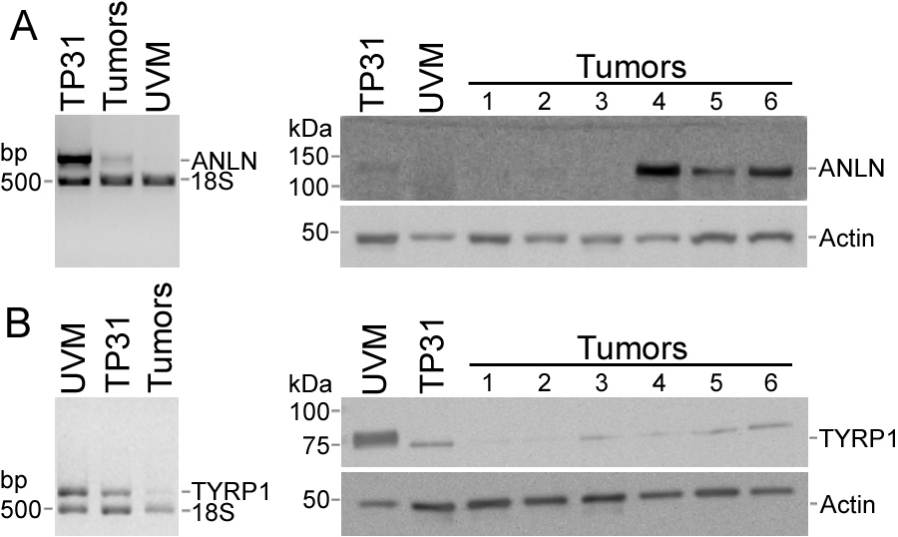
*ANLN* and *TYRP1* expression in UM. **A**: The expression level of *ANLN* was measured by semi-quantitative RT–PCR (left panel) and western blot (right panel) in the TP31 cell line, UM primary tumors (Tumors), and UVM. **B**: The expression level of *TYRP1* was measured by semi-quantitative RT–PCR (left panel) and western blot (right panel) in UVM, the TP31 cell line and UM primary tumors (Tumors). The 18S rRNA was used as an internal control of amplification (489 bp). Actin was used as a protein loading control. Data are representative of three independent experiments.

**Figure 4:**
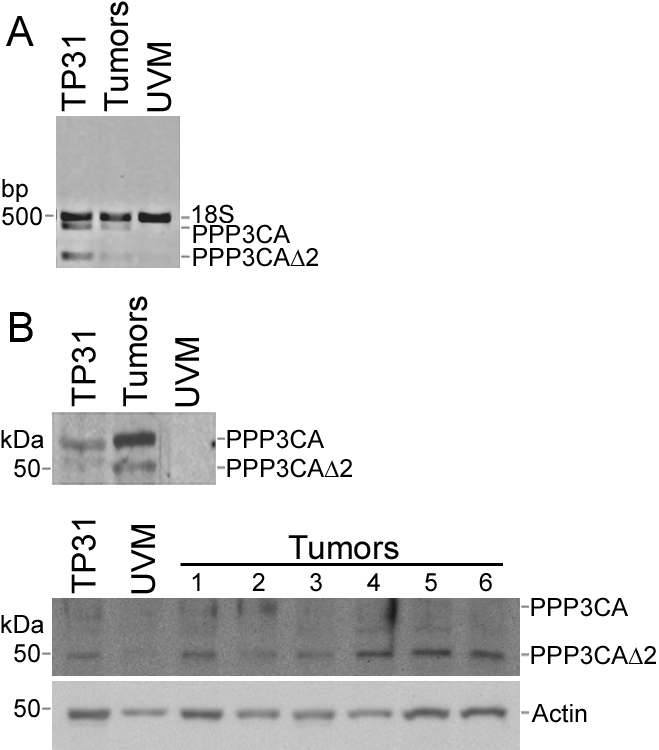
*PPP3CA* expression in UM. **A**: The expression level of *PPP3CA* mRNA was measured by semi-quantitative RT–PCR in the TP31 cell line, UM primary tumors (Tumors), and UVM. **B**: The expression level of the PPP3CA protein was measured by western blot in the TP31 cell line, UVM, and UM primary tumors (left panel: pool of protein extracts from UM primary tumors; right panel: individual UM primary tumors). A new splice variant was detected, which lacks parts of the NH_2_-terminal and catalytic domains after the deletion of exon 2 (PPP3CAΔ2). The 18S rRNA was used as internal control of amplification (489 bp). Actin was used as a protein loading control. Data are representative of three independent experiments.

Semi-quantitative RT–PCR analyses were performed to compare the expression of the upregulated genes acidic nuclear phosphoprotein 32 member E (*ANP32E*), *CKAP5*, and denticleless homolog (*DTL*) from the TP31 cell line subtracted library (Figure 2A), as well as the downregulated genes *CTSK*, methylthioadenosine phosphorylase (*MTAP*), and testis-specific protein-like 1 y-encoded (*TSPYL5*) from the UVM subtracted library (Figure 2B) between the TP31 cell line, UM primary tumors, and UVM. These genes were chosen for their redundancy in the SSH libraries and/or their implication in malignant melanoma or other cancers, after microarray validation. These analyses showed identical expression patterns as those revealed by the SSH technique, indicating that the SSH data were accurate. Indeed, no band could be seen in the UVM for the upregulated genes (Figure 2A). In addition, no band could be observed in the TP31 cell line and UM primary tumors for the downregulated genes (Figure 2B). We next examined both mRNA and protein expression of *ANLN* and *TYRP1* between TP31 cell line, UM primary tumors, and UVM (Figure 3). Neither mRNA nor protein could be detected for *ANLN* in UVM compared to TP31 and some primary tumors (Figure 3A). *TYRP1* mRNA and protein level were greatly decreased in TP31 cell line and primary tumors compared to UVM (Figure 3B). In addition, *PPP3CA* mRNA or protein was not detected in UVM compared to TP31 and primary tumors (Figure 4). Moreover, we identified a new splice variant of *PPP3CA* in the TP31 cell line as well as in the primary tumors. This shorter amplicon was not expressed by the UVM (Figure 4A, lower band). The sequencing of this amplicon allowed the identification of a new splice variant of *PPP3CA* lacking exon 2 (*PPP3CAΔ2*). Western blots were performed to confirm the existence of a splice variant of the PPP3CA protein using an antibody raised against exon 1 to assess the expression of both the native protein and the splice variant lacking exon 2. As can be seen in Figure 4B (left panel), two protein products were detected at 51- and 59-kDa in the extracts prepared from both the TP31 cell line and a pool of UM primary tumor protein extracts, but not in UVM. When using several UM primary tumors separately, some expressed both the native PPP3CA protein and the splice variant, while others expressed only the splice variant (Figure 4B, right panel).

## Discussion

Only a few oncogenes involved in the development of UM primary tumors have been discovered until now. This study was undertaken to identify additional genes that could be required for malignant transformation of melanocytes by preparing subtracted libraries using normal UVM and the UM cell line TP31. The sequencing of upregulated cDNAs from the TP31 cell line subtracted library and downregulated cDNAs from the UVM subtracted library has resulted in the identification of 73 genes differentially expressed by UM.

Many genes found to be upregulated in the TP31 cell line subtracted library were previously associated with cancer, including *ANLN* and *PPP3CA*. ANLN is an actin binding protein that can associate with septins and is involved in cytokinesis [15]. Several studies have demonstrated an overexpression of *ANLN* in cancer such as in several of the UM tumors assayed in the present study. Indeed, Hall et al. assessed *ANLN* expression in several human tissue samples and cell lines by microarray analyses and demonstrated that the median level of *ANLN* expression was higher in tumors than in normal tissues and correlated with the metastatic potential of these tumors [16]. Moreover, *ANLN* overexpression was shown to increase levels of active RhoA and subsequently cell motility [16]. Therefore, given that increased genomic instability is a feature of metastatic UM tumors [1], *ANLN* overexpression could create defects in cytokinesis leading to binucleation and genomic instability. Calcineurin is a protein phosphatase regulating the Ca^2+^/calmodulin complex, formed by a catalytic A subunit (PPP3CA) and a regulatory B subunit [17]. Whereas interest was previously confined to its activity in immune response, calcineurin is now becoming recognized as a predominant participant in oncogenesis [18]. Indeed, the Ca^2+^/calcineurin/ nuclear factor of activated T-cells (NFAT) signaling pathway influences different aspects of tumor biology, such as stimulation of angiogenesis through upregulation of vascular endothelial growth factor (VEGF), tumor cell proliferation through upregulation of myelocytomatosis oncogene (MYC), and tumor cell migration through cyclooxygenase-2 (COX-2) [18]. Native PPP3CA contains five distinct domains: the NH_2_-terminal, the catalytic, the B-subunit binding, the calmodulin-binding and the auto-inhibitory domains [17]. Three variants were identified previously and one splice variant showed no phosphatase activity, but was able to stimulate the phosphatase activity of the native protein, thus rendering PPP3CA more calcium-sensitive [19]. In the present study, we reported the expression of a novel PPP3CA splice variant in primary UM cells that lacks part of the NH_2_-terminus and catalytic domains (PPP3CAΔ2; GenBank#AY904364; Figure 4). Further biochemical analysis will be necessary to determine whether or not the catalytic activity of this new variant is altered.

Among the genes found to be downregulated in the UVM subtracted library, several were previously associated with melanoma, such as melanocytic markers *TYRP1*, *EDNRB*, *MTAP*, and sex determining region Y box 4 (*SOX4*; Table 4). Indeed, the expression of *TYRP1* was inversely correlated with tumor stage in malignant melanoma [20]. A decreased expression of *EDNRB* was previously associated with early metastasis and short survival in UM [21]. A tissue microarray study in malignant melanoma demonstrated a significant reduction of *MTAP* in melanomas and metastases compared with nevi [22]. In addition, *SOX4* expression was reduced in metastatic malignant melanoma compared with dysplastic nevi and primary melanoma. *SOX4* downregulation was correlated with a poor prognosis and *SOX4*-depleted melanoma cells showed enhanced invasion and migration [23].

SSH technique was previously used in efforts to identify genes that were differentially expressed in UM [21,24,25]. One study was aiming to identify genes involved in the development of metastases [21] whereas the other studies were rather aiming to identify genes that are involved in malignant transformation of melanocytes [24,25]. Only a very small number of genes were reported by these studies as a result of the analysis of their subtracted library [21,24,25]. As a consequence, an extensive comparison between our results and their data could not be performed. Among the four genes they reported, namely the cysteine-rich protein 61 (*CYR61*) and tissue factor (also called coagulation factor III), *EDNRB* and the AXL receptor tyrosine kinase (*AXL*), only *EDNRB* was identified in our UVM subtracted library [21,24,25].

One limitation of the present study is the use of only one primary UM cell line for the preparation of the SSH libraries. However, the validation of the SSH list of genes by microarray, semi-quantitative RT–PCR and western blot with primary tumor samples compensated for the use of only the TP31 cell line as starting material. It is noteworthy that the TP31 cell line is devoided of the *GNAQ/GNA11* mutations and that, consequently, the genes reported in the present study could allow to establish a new mechanism for early gene expression changes leading to malignant transformation and proliferation of uveal melanocytes. Consistent data have been obtained when comparing the SSH results with microarray data and RNA/protein extracts of primary UM tumors, suggesting that UM cell lines could represent good models for such analyses given the limited availability of primary UM tumor samples. Studying the function of these genes and their biologic pathways may lead to the development of new therapeutic options.
